# Influence of Dissolved Oxygen Level on Chitin–Glucan Complex and Mannans Production by the Yeast *Pichia pastoris*

**DOI:** 10.3390/life12020161

**Published:** 2022-01-21

**Authors:** Inês Farinha, Sílvia Baptista, Maria A. M. Reis, Filomena Freitas

**Affiliations:** 1 Pharma 73, S.A., Edifício Arcis, Rua Ivone Silva, 6, 4º Piso, 1050-124 Lisboa, Portugal; ines.farinha@pharma73.com; 2 UCIBIO-Applied Molecular Biosciences Unit, Department of Chemistry, School of Science and Technology, NOVA University Lisbon, 2819-516 Caparica, Portugal; silvia.baptista@73100.pt (S.B.); amr@fct.unl.pt (M.A.M.R.); 3 Associate Laboratory i4HB-Institute for Health and Bioeconomy, School of Science and Technology, NOVA University Lisbon, 2819-516 Caparica, Portugal; 4 73100 Lda., Edifício Arcis, Rua Ivone Silva, 6, 4º Piso, 1050-124 Lisboa, Portugal

**Keywords:** chitin–glucan complex (CGC), mannans, *Pichia pastoris*, dissolved oxygen (DO) level, glycerol

## Abstract

The yeast *Pichia pastoris* was cultivated under different dissolved oxygen (DO) levels (5, 15, 30 and 50% of the air saturation) to evaluate its impact on the production of the cell-wall polysaccharide chitin–glucan complex (CGC) and mannans. Decreasing the DO level from 50 to 15% had no significant impact on cell growth but substrate conversion into biomass was improved. Under such conditions, a mannans content in the biomass of 22 wt% was reached, while the CGC content in the biomass was improved from 15 to 18 wt%, confirming that the DO level also impacted on *P. pastoris* cell-wall composition. Overall mannans and CGC volumetric productivity values of 10.69 and 8.67 g/(L. day) were reached, respectively. On the other hand, the polymers’ composition was not significantly affected by decreasing the DO level. These results demonstrated that considerable energy savings can be made in the polysaccharide production process by reducing the DO level during cultivation of *P. pastoris* by improving the overall polymers’ productivity without altering their composition. This has impact on the polysaccharide production costs, which is of considerable relevance for process scale-up and products’ commercialization.

## 1. Introduction

Chitin–glucan complex (CGC) is a co-polymer composed of chitin and β-(1,3)-glucans. The combination of the two bioactive polysaccharides in the same macromolecule makes CGC a promising biomaterial for several medical applications, due to its immunomodulator, antitumor, antioxidant and antimicrobial properties [1]. Recently, CGC has been used in pharmaceutical applications, such as the treatment of diabetes, obesity [2], heart diseases [3] and wound healing [4], being also proposed for use in other areas, such as cosmetics or wine clarification [1].

Mannans are polysaccharides mainly composed of mannose units. Similarly to CGC, mannans are also an interesting material in the medical field, due to their immunological, antimutagenic and antioxidant properties [5]. Moreover, mannans can also be used in food formulations due to their emulsifying and prebiotic effects [6].

CGC and mannans represent 10–30 wt% and 7–13 wt% of the cell wall of several yeasts and fungi, respectively [7]. The yeast cell wall is a dynamic structure mainly composed of polysaccharides (chitin, glucans and mannans) and proteins that confer rigidity and stability to the cells in response to several environmental stress factors [8]. The yeast cell-wall composition can be affected by several factors, including the culture medium, temperature, external pH, oxygen level and hypo-osmotic stress [9]. To cope with adverse conditions and maintain cell viability, yeast cells activate the cell-wall integrity (CWI) pathway that results, for example, in increased production of some cell-wall components, such as chitin and mannoproteins, and/or in the reorganization of certain covalent linkages between the cell-wall components [9].

*Pichia pastoris* (also known as *Komagataella pastoris*) is a methylotrophic yeast widely used for the production of recombinant proteins [10]. Due to its capacity to achieve high cell densities and high cell growth rates, *P. pastoris* is also an interesting source of CGC and mannans [7]. Moreover, *P. pastoris* has the ability to grow over a wide range of pH values (3.0–7.0), temperatures (20–30 °C) and dissolved oxygen (DO) levels (10–50%) [11]. Despite the high cell titer (above 100 g/L) and high protein productivity achieved in most fermentation processes, little is known about the real impact of the fermentation conditions on the yeast cell-wall composition and, specifically, on CGC and mannans contents. According to Chagas et al. [12], the chitin content in CGC is reduced to below 14 mol% by cultivating *P. pastoris* out of pH and temperature ranges of 4.5–5.8 and 26–33 °C, respectively. Gmeiner et al. [13] also noticed slight changes on recombinant *P. pastoris* CBS7435 cell morphology by changing the pH, temperature and DO level. Nevertheless, the impact of DO level on yeast cell-wall composition remains unrevealed, especially for *P. pastoris* strains.

In this work, the influence of the DO level on CGC and mannans content in *P. pastoris* cells and the polymers’ composition were evaluated. Batch bioreactor cultivations under different DO levels were conducted. Afterward, a fed-batch experiment was performed under the DO level that led to the highest biomass and polymer production to validate such conditions in a high cell density cultivation.

## 2. Materials and Methods

### 2.1. Yeast Strain and Culture Medium

*Pichia pastoris* strain DSM 70877 was used in all experiments. *P. pastoris* was cultivated in standard basal salts medium (BSM) (Pichia Fermentation Process Guidelines, Invitrogen), as described by Farinha et al. [14]. BSM was supplemented with glycerol (86–88 wt%) to give a concentration of ≈50 g/L.

### 2.2. Batch Bioreactor Cultivations

The inoculum for each bioreactor cultivation was prepared by inoculating 1 mL of the cryopreserved (at −80 °C) culture in 200 mL BSM and incubating for 40 h, at 30 °C and 200 rpm. The inoculum thus obtained was used to inoculate a 2 L bioreactor (BioStat B-Plus, Sartorius, Göttingen, Germany), with an initial working volume of 1.4 L. All the experiments were performed with controlled temperature (30 ± 0.1 °C) and pH (5.0 ± 0.02). The pH was automatically controlled by the addition of 25% (*v*/*v*) ammonium hydroxide solution that also served as the nitrogen source, and a hydrochloric acid 2 M solution. The air flow rate was kept constant at 1.4 SLPM (standards liters per minute). The DO level was controlled by the automatic variation of the stirrer speed (300–2000 rpm). Batch experiments were performed with DO levels of 5, 15, 30 and 50% of the air saturation, for 42 h. Samples (12 mL) were periodically withdrawn from the bioreactor, for quantification of the dry cell weight (DCW), glycerol, ammonium, CGC and mannans.

### 2.3. Fed-Batch Bioreactor Cultivation

For the fed-batch bioreactor experiment, an inoculum was prepared by inoculating 4 mL of the cryopreserved (at −80 °C) culture in 800 mL of BSM and incubation for 40 h, at 30 °C and 200 rpm. This inoculum was used to inoculate a 10 L bioreactor (BioStat B-Plus, Sartorius), with an initial working volume of 8 L. The bioreactor was operated as described above for the batch experiments, except for the DO level that was controlled at 15% of the air saturation, by an automatic cascade comprising the variation of the stirrer speed (300–1000 rpm) and supplementation of the air stream with oxygen that was triggered when the maximum stirring rate was not enough to maintain the DO level at the set point. 

The experiment started with a 24 h batch phase, followed by a 23 h fed-batch phase, wherein a feed solution was added, as described by Farinha et al. [14]. The feeding solution was composed of glycerol (86–88 wt%) supplemented with Pichia trace mineral (PTM) solution in a proportion of 24 mL of PTM solution per liter of glycerol. The feeding profile was set in the control unit of the bioreactor, which automatically fed the reactor with substrate at a rate that gradually increased from 6.6 to 7.6 g/(L. h) (considering the initial working volume of the reactor), giving an overall feeding of 1359 g of glycerol. Samples (20 mL) were periodically withdrawn from the bioreactor, for quantification of the DCW, glycerol, ammonium, CGC and mannans.

### 2.4. Analytical Techniques

For determination of the DCW, 4 mL fermentation broth samples were centrifuged (8000× *g*, 10 min). The pellet was used for the gravimetric quantification of biomass, while the cell-free supernatant was used for glycerol and ammonium quantification. The gravimetric quantification of DCW was made as described by Farinha et al. [7]. 

For glycerol quantification, the cell-free supernatant was analyzed by high-performance liquid chromatography (HPLC). The analysis was performed as described by Farinha et al. [7]. Briefly, the analysis was performed with a MetaCarb 87H column (Varian Inc., Palo Alto, CA, USA) and a differential refractometer RI-71 detector (Merck, Darmstadt, Germany). The samples were analyzed at 50 °C, using H2SO4 0.01 N as eluent, at a flow rate of 0.6 mL/min. Glycerol 86–88 wt% (Scharlab, Barcelona, Spain) was used as standard, at a concentration range of 0.065–1.0 g/L. The samples were diluted to have their concentration below 1.0 g/L.

For ammonium quantification, the cell-free supernatant was analyzed as described by Farinha et al. [7]. An ammonium chloride solution was used as standard, at a concentration range of 4.0–20.0 ppm. The samples were diluted to have their concentration below 20 ppm.

### 2.5. Polymers’ Extraction

For polymer extraction from *P. pastoris* biomass, 100 mg of dried biomass samples were resuspended in 30 mL NaOH 5 M and subjected to a temperature of 65 °C, for 2 h. After the chemical treatment, the suspension was centrifuged (8000× *g*, 10 min), for separation of the alkaline insoluble material (AIM) from the alkaline soluble material (ASM). CGC was obtained from the AIM, as described by Farinha et al. [14]. 

To obtain the mannan samples, the ASM was dialyzed with a 12,000 MWCO membrane (Nadir^®^—dialysis tubing, Carl Roth, Karlsruhe, Germany) against deionized water, for 48 h. The deionized water was changed twice a day, until a conductivity below 10 μS/cm was achieved. The dialyzed samples were freeze dried for the gravimetric quantification of mannans.

### 2.6. Polymers’ Characterization

For CGC sugar compositional analysis, dried samples were subjected to two acid hydrolyses, as described by Farinha et al. [14]. The glucose monomers of the β-glucan fraction were obtained by hydrolyzing the CGC samples with trifluoroacetic acid (TFA) 99% (at 120 °C, for 2 h). The glucosamine monomers, present in the chitin fraction, were quantified after hydrolyzing the CGC samples with hydrochloric acid (HCl) 4 M (at 120 °C, for 5 h). The mannans’ sugar composition was also performed with the same TFA hydrolysis procedure. The hydrolysates were analyzed by HPLC, as described by Farinha et al. [7]. Glucose, mannose and glucosamine (Sigma-Aldrich, Saint Louis, MO, USA) were used as standards, at concentrations ranging between 0.005 and 0.1 g/L and were subjected to the same hydrolysis procedures as the polysaccharide samples. 

For determination of the protein content, dried polysaccharide samples (7 mg) were treated with NaOH 2 M (1 mL) in sealed glass vials, at 120 °C, for 15 min. The supernatant obtained by centrifugation (10,000× *g*, 10 min) was used for the protein assay, according to the modified Lowry method, as described by Farinha et al. [7]. Bovine serum albumin (BSA, Sigma-Aldrich) was used as standard, at concentrations of 0–6.0 mg/mL. The inorganic salts content was evaluated by pyrolysis decomposition of the samples (≈50 mg) at a temperature of 550 °C, for 24 h.

### 2.7. Kinetic Parameters

The maximum specific cell growth rate (μ_max_, h^−1^) was determined using the following equation (Equation (1)):

(1) $$\ln\left(\frac{X}{X_{0}}\right){=\mu }_{\max}\;t$$

where X and the X_0_ are the DCW (g/L) at time t and at time t = 0, respectively.

The biomass, CGC and mannans yields, Y_X/S_ (gx/gs), Y_CGC/S_ (gc/gs) and Y_M/S_ (gm/gs) were determined by Equations (2)–(4), respectively:

(2) YXS=ΔXΔS

(3) YCGCS=ΔCGCΔS

(4) YMS=ΔMannansΔS

where ∆X is the biomass (g) produced during the experiment, ∆CGC and ∆Mannans are the CGC and mannans produced (g), respectively, and ∆S is the total glycerol consumed during the same time interval (g).

The CGC and mannans volumetric productivity, r_CGC_ (g/(L. day)) and r_M_ (g/(L. day)), respectively, were determined using Equations (5) and (6):

(5) $$r_{\mathrm{CGC}}=\frac{\Delta \mathrm{CGC}}{t}$$

(6) $$r_{M}=\frac{\Delta \mathrm{Mannans}}{t}$$

where ∆CGC and ∆Mannans are the CGC and mannans produced (g/L) at time t (day).

### 2.8. Statistical Analysis

The comparison of means between DO level and each kinetic parameter (Table 1) was carried out by an independent sample *t*-test, using the software Statistica 7.0 (StatSoft Inc., Tulsan, OK, USA). All the factors were evaluated by the *p*-value at a 95% confidence level.

## 3. Results

### 3.1. Effect of the DO Level on P. pastoris Cultivation

#### 3.1.1. Biomass Production

Batch bioreactor fermentations were performed with the DO level controlled at 5, 15, 30 or 50% of the air saturation. The specific cell growth rate was significantly similar (0.15–0.17 h^−1^) for all assays, except for the lowest DO level tested (5%), in which the culture grew at a lower value, 0.12 h^−1^ (Table 1). An overall biomass production of 25.71 g/L was obtained by cultivation with a DO level of 15%, while lower DCW values (22.51–23.29 g/L) were achieved for higher or lower DO levels (Table 1). The decrease of the DO level from 50 to 15% resulted in a 15% improvement of the biomass production. However, this higher biomass production was not significantly different, being statistically similar between the different DO levels studied (*p*-value > 0.05). Glycerol was completely consumed in all experiments, taking around 25–29 h for its depletion. However, a lower glycerol consumption rate was observed for the experiment performed at the lowest DO level of 5%, in which substrate exhaustion took around 40 h. The highest biomass yield, 0.45 gx/gs, was also achieved in the experiment performed with a DO level of 15%, with statistical significance (*p*-value < 0.05) (Table 1). In opposition, fermentations with DO levels of 5 and 50% resulted in significantly lower biomass yields (0.36–0.41 gx/gs). In all experiments, no ammonium depletion was observed, being between 5 and 9 g/L during most of the time of the batch process, in all the experiments performed for this study.

#### 3.1.2. CGC Production

As shown in Table 1, a CGC content of 18 wt% was obtained for the experiment performed at a DO of 5%, while at higher DO levels (50%), the CGC content was 15 wt%. However, the highest CGC production, 4.42 g/L, was achieved in the experiment performed at a DO level of 15%, which also resulted in the highest CGC volumetric productivity, 2.51 g/(L. day) (Table 1). The lowest productivity of 2.09 g/(L. day) was observed for the cultivation with a DO level of 50% (Table 1). Regarding the CGC production, the decrease of the DO level from 50 to 15% improved the CGC production by 24.5%. However, this increase on CGC content and production was also not statistically significant (*p*-value > 0.05). The CGC yield on a substrate basis was also significantly higher for the experiment performed with a DO of 15% (0.08 gc/gs), compared to the other DO levels tested. On the other hand, the CGC composition shows a similar chitin/β-glucan molar ratio, between 11:89 and 13:87, for the co-polymers obtained in all batch experiments.

#### 3.1.3. Mannans Production

Similarly, mannans production was also higher for the experiment performed at a DO level of 15% (5.32 g/L), coincident with the highest mannans content in the biomass of 20 wt%, among the tested conditions (Table 1). The 5% difference in mannans content, between the DO levels of 50 and 15% resulted in an improvement of 54% in the mannans production, when the DO level in the medium decreases from 50 to 15%. Similar with biomass and CGC production, the mannans production was also not statistically significant (*p*-value > 0.05). The batch experiment with the DO level of 15% was also the one with the highest mannans yield and volumetric productivity, 0.09 gm/gs and 3.02 g/(L. day), respectively. Interestingly, with the DO level of 15%, mannans production was higher than that of CGC (Table 1), with an overall cell-wall polysaccharide content in the biomass of 37 wt%. The mannans samples were mainly composed of mannose units (82–85 wt%), with traces of glucose (2.1–5.3 wt%) and glucosamine (3.0–4.4 wt%). These samples also contained a residual inorganic salts content of 0.3–1.5 wt%, while the protein content was 7.6–9.0 wt%. 

### 3.2. Fed-Batch Bioreactor Cultivation

**Figure 1:**
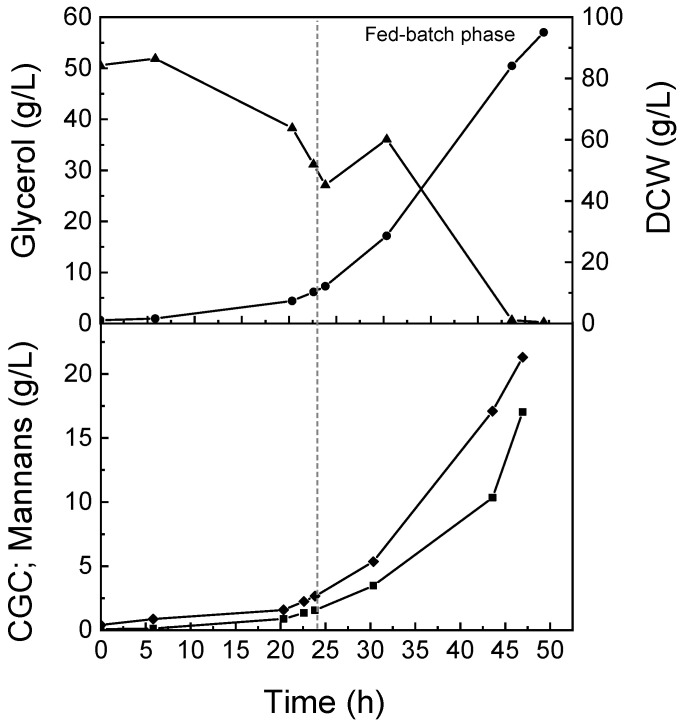
Cultivation profiles obtained in fed-batch experiment with *P. pastoris* DSM 70877: ▲, glycerol concentration; ●, DCW; ■, CGC; ◆, mannans.

This cultivation included a batch phase that lasted 24 h, followed by a 23 h fed-batch phase (Figure 1). The culture grew at a maximum specific cell growth rate of 0.15 h^−1^. A cell concentration of 95.1 g/L was reached at the end of the run (47 h) (Figure 1). All the glycerol supplied to the culture was completely consumed during the fed-batch run, with an average specific consumption rate of 0.21 gs/(gx. h). There was an overall glycerol consumption of 1771.6 g, corresponding to a biomass yield of 0.51 gx/gs (Table 2). The ammonium level in the medium was kept between 2.2 and 5.2 g/L during the entire experiment, since an ammonia solution was supplemented to the medium for pH control. At the end of the run, the biomass had CGC and mannans content of 18 and 22 wt%, respectively (Table 2). The corresponding overall CGC production and volumetric productivity values were 17.04 g/L and 8.71 g/(L. day), respectively (Table 2). The co-polymer’s yield was 0.09 gc/gs. The mannans production (21.3 g/L) was higher than that of CGC (17.04 g/L) (Table 2). The mannans yield on a substrate basis was 0.11 gm/gs (Table 2). The chitin/β-glucan molar ratio of the CGC produced in the fed-batch experiment was 16:84. The mannans produced in this experiment were also mainly composed of mannose units (82 wt%), with traces of glucose and glucosamine (2.0 and 2.9 wt%, respectively). The sample also contained low inorganic salts content (1.3 wt%) and a protein content of 12.3 wt%. 

## 4. Discussion 

### 4.1. Effect of the DO Level on P. pastoris Cultivation

#### 4.1.1. Biomass Production

Batch cultivations were performed with the DO level controlled at 5, 15, 30 or 50% of the air saturation, to evaluate the impact on both *P. pastoris* cell growth and polymer production and composition. The specific cell growth rate observed in these experiments (0.15–0.17 h^−1^) were within the range of the values reported for *P. pastoris* DSM 70877 cultivation at a DO level of 50%, under similar batch conditions (0.15–0.16 h^−1^) [14,17]. Similar specific cell growth rates (0.14–0.16 h^−1^) were also reported for other *Pichia pastoris* strains, using DO levels of 20–30%. For example, Robert et al. [15] and Noseda et al. [16] obtained specific cell growth rates of 0.14–0.19 h^−1^ for *P. pastoris* X-33 and GS115 during batch-phase processes, respectively, also using glycerol as a carbon source and similar operation conditions.

However, the maximum biomass concentration was achieved with a DO level of 15% (25.71 g/L). Glycerol was completely consumed in all experiments, but at DO level of 5%, its consumption rate was lower than that observed for the other DO levels tested. These results are in accordance with the lower specific cell growth rate observed for the same experiment (Table 1). Cultivation with a DO level at 15% also resulted in the highest biomass yield (0.45 gx/gs), while with DO levels of 5 and 50%, a less efficient conversion of glycerol into biomass was observed (Table 1). The low biomass yield observed for DO level of 50% was probably due to substrate oxidation [18]. On the other hand, at the DO level of 5%, the production of by-products (such as organic acids or ethanol that were detected at low levels) was observed, which has probably contributed to the lower biomass yield reached in this experiment. Such by-products were not detected for the remaining experiments conducted at higher DO levels. These results suggest that cultivation of *P. pastoris* under a DO level of 15% provides a more efficient conversion of the substrate into biomass. A similar trend was reported for several *P. pastoris* cultivations at DO levels between 10 and 30%. For example, Günes et al. [19] and Luo et al. [20] reported a biomass production of 32–44 g/L, for biomass yields of 0.48–0.53 gx/gs in fermentation processes with a DO level of 20%, for recombinant *P. pastoris* strains X-33.

#### 4.1.2. CGC Production

The CGC content in *P. pastoris* biomass observed in the batch experiments was enhanced by decreasing the DO level, from 50 to 5% (Table 1). Farinha et al. [14] and Araújo et al. [17] reported CGC contents in the biomass of 13–15 wt% in *P. pastoris* cultivations under similar conditions, but with DO levels of 50%. These results suggest that imposing a lower oxygen level may have influenced cell-wall polysaccharides’ synthesis. It was reported that environmental (pH, DO level or temperature) or chemical induced factors (medium composition) can trigger cell-wall compensatory mechanisms to protect the yeast cells, keeping their integrity [9]. Those response mechanisms are the so-called cell-wall integrity (CWI) pathway, responsible for the biogenesis and organization of the cell wall [8,9]. These cell-wall response signals are characterized by alterations of cell-wall composition and morphology [21]. Despite the differences observed in CGC production for different DO levels, the CGC content in the biomass achieved in all experiments (15–18 wt%) is within the range of values reported for the cell-wall polysaccharide content of several yeast and fungi strains (5–30 wt%) [22,23,24]. For example, Nawawi et al. [22] reported a CGC content between 15 and 25 wt% for *Agaricus bisporus* and similar values (21–22 wt%) were also obtained for *Schizophyllum commune* biomass [24]. 

Despite not being the DO level condition with the highest CGC content in biomass, the 15% DO level enabled the highest CGC production (4.42 g/L) and productivity (2.51 g/(L. day)), due to the higher biomass production. Comparing with the CGC production obtained with the DO level at 50%, this lower DO level represents an improvement of 24.5% on CGC production. The CGC yield on a substrate basis was also the highest, 0.08 gc/gs, meaning that at this DO level, the glycerol consumed is more efficiently converted into CGC, similarly to what was observed for biomass yield.

Regarding the CGC composition obtained in the batch experiments, a clear impact was not observed on its chitin/β-glucan molar ratio that ranged between 11:89 and 13:87. These ratios are also within the values reported for *P. pastoris* CGC, between 11:89 and 22:78 [7,14]. 

#### 4.1.3. Mannans Production

The influence of DO level on *P. pastoris* mannans production was also studied. The DO level of 15% also revealed to be the best oxygen level condition for mannans production, since it enabled the highest polysaccharide content (20 wt%) and concentration (5.32 g/L) (Table 1). Lower values (4–18 wt%) have been reported for mannans production by other yeasts and fungi [25,26,27], which demonstrates the advantage of using *P. pastoris* as a yeast mannans producer. Galinari et al. [25], for example, obtained 13 wt% of mannans content from *Kluyveromyces marxianus* biomass. A mannoprotein content of 12–18 wt% was also reported by Faustino et al. [26] for *Saccharomyces cerevisiae*. This higher mannans production observed at the 15% DO level was also reflected in the highest mannans productivity (3.02 g/(L. day)) and mannans yield (0.09 gm/gs) (Table 1).

The higher mannans production, combined with the CGC production achieved with the 15% DO level, resulted in an overall polysaccharide content in the biomass of 37 wt%. This value is higher than the 27–32% reported for *Aspergillus oryzae* [28], confirming the high potential of *K. pastoris* to be used in cell-wall polysaccharide production processes. This enhancement in overall polysaccharide content in *P. pastoris* cell walls confirmed that the lower DO level impacted on cell-wall composition. Moreover, the major influence was observed in the mannans layer (outer cell wall), where a higher difference between the mannans content in biomass was observed (15–20 wt%) in the batch experiments performed (Table 1). As it was observed for CGC (in the inner cell wall), the mannoprotein expression is also tightly regulated by the oxygen level [9]. 

Similarly to CGC, the mannans composition was also not significantly affected by the DO levels tested, namely, its sugar composition and inorganic salts and proteins content. The presence of protein and other sugars in mannans fractions extracted from yeasts is common. For example, the mannans extracted from *K. marxianus* had a protein content up to 7 wt% [25]. The mannoprotein fraction extracted from *this yeast* also revealed the presence of 10 wt% of glucans. Despite no considerable differences being detected in mannans protein content, it was also reported in other studies that its composition can also be influenced by low oxygen levels [8,9]. 

Given these findings, cultivation of *P. pastoris* at a DO level of 15% seems to be the most adequate condition for the production of both CGC and mannans, with high productivities, without any significant impact on the polymers’ composition.

### 4.2. Fed-Batch Bioreactor Cultivation

Aiming to validate the DO level of 15% as the best for production of CGC and mannans, a fed-batch bioreactor cultivation was performed. The maximum specific growth rate obtained during the batch phase was similar to that of the batch experiment performed with the same DO level, 0.14–0.15 h^−1^ (Table 1). A high cell density cultivation was obtained after the 47 h of the run, 95 g/L (Figure 1). The average glycerol specific consumption rate (0.21 gs/gx. h) was higher than the values reported for some *P. pastoris* strains, such as the CBS7435 strain, which had specific glycerol consumption rates of 0.12–0.13 gs/gx.h for cultivations under a similar DO level [13]. The biomass yield of 0.51 gx/gs represents an improvement on biomass yield compared to the value reported by Farinha et al. [14], 0.49 gx/gs, for cultivation under a DO level of 50% (Table 2). Similar biomass production results were also obtained for the cultivation of recombinant *P. pastoris* strains X-33 and GS115 that achieved DCW values of 70.0 and 102 g/L, respectively, and biomass yields of 0.50 and 0.57 gx/gs, respectively (Table 2), under DO levels of 30% [15,16]. 

CGC production at a DO level of 15% was also improved in this fed-batch experiment compared to previous reports for cultivation of *P. pastoris* with a higher DO level (50%). In fact, the CGC volumetric productivity was improved by around 13%, from 7.70 to 8.71 g/(L. day) [14]. Similarly to the batch experiment performed with the same DO level, the mannan production was also slightly higher than the CGC production obtained in the same batch experiment (Table 1 and Table 2). The mannan yield on a substrate basis was also increased to 0.11 gm/gs (Table 2), compared with the 0.09 gm/gs obtained in the batch experiment (Table 1).

The CGC obtained in this fed-batch cultivation had a higher chitin content (16 mol%) compared with the CGC obtained in the batch studies (11:89–13:87), but still within the range of values previously reported, 11:89–22:78 [7,14]. No major differences were observed for the mannans produced in this experiment compared with the mannans produced in the batch cultivations, with the exception of a relatively higher protein content (12 wt%). 

The results obtained in the fed-batch experiment at a DO level of 15% demonstrated that the lower DO level influenced *P. pastoris* cell-wall composition, by enhancing the CGC and mannans contents, and by increasing the chitin fraction of the CGC co-polymer and the protein content in mannans. These findings are in agreement with several yeast cell-wall studies, that referred that stress factors such as temperature or oxygen levels regulate the polysaccharide expression, mainly by changing the protein composition in the outer cell wall and increasing the chitin content in the inner cell wall [8,9]. 

On the other hand, lowering the DO level during the fermentation operation also enabled considerable energy savings in this bioprocess, without compromising the overall product productivity and polymers’ composition. CGC and mannans can be used as sources of two monosaccharides with high market value, namely, glucosamine and mannose. Therefore, increasing their content in *P. pastoris* cell walls may be advantageous if the production of these sugars is envisaged.

## 5. Conclusions

This work demonstrated that reducing the DO level from 50 to 15% during cultivation of *P. pastoris* provided a more efficient conversion of glycerol into biomass, as demonstrated by the higher growth yield obtained. More importantly, the production of the cell-wall polysaccharide CGC and mannans was also improved. The contents of both polymers in the biomass were increased, which resulted in higher production and volumetric productivities. In particular, CGC productivity was improved by 13% without any significant impact on its composition. Thus, reducing the DO level during cultivation has a positive impact on the process productivity while enabling considerable bioprocess energy and cost savings regarding the oxygen demand.

## Figures and Tables

**Table 1:** Results obtained in the DO batch experiments for *P. pastoris* growth and polymer production: maximum specific cell growth rate (μ_max_); dry cell weight (DCW); overall CGC (r_CGC_) and mannans (r_M_) volumetric productivities; yields of CGC (Y_CGC/S_) and mannans (Y_M/S_) on a substrate basis.

DO (%)	μ_max_ (h^−1^)	DCW (g/L)	CGC Content (wt%)	CGC (g/L)	Mannan Content (wt%)	Mannans (g/L)	r_CGC_ (g/(L. day))	r_M_ (g/(L. day))	Y_X/S_ (g_x_/g_s_)	Y_CGC/S_ (g_c_/g_s_)	Y_M/S_ (g_m_/g_s_)
5	0.12	22.51	18	4.16	17	3.92	2.36	2.23	0.38	0.07	0.07
15	0.15	25.71	17	4.42	20	5.32	2.51	3.02	0.45	0.08	0.09
30	0.16	23.29	17	4.16	16	3.80	2.37	2.16	0.41	0.07	0.07
50	0.17	22.37	15	3.55	15	3.47	2.09	2.04	0.36	0.06	0.06
*p*-value	0.044	0.881	0.431	0.076	0.440	0.077	0.060	0.060	0.046	0.044	0.044

**Table 2:** Results obtained in the fed-batch experiment for *P. pastoris* biomass and polymer production and comparison with the literature: dry cell weight (DCW); overall CGC (r_CGC_) and mannans (r_M_) volumetric productivities; yields of biomass (Y_X/S_), CGC (Y_CGC/S_) and mannans (Y_M/S_) on glycerol basis.

Strain	DO (%)	DCW (g/L)	CGC (wt%)	CGC (g/L)	Mannans (wt%)	Mannans (g/L)	r_CGC_ (g/(L. day))	r_M_ (g/(L. day))	Y_X/S_ (g_x_/g_s_)	Y_CGC/S_ (g_c_/g_s_)	Y_M/S_ (g_m_/g_s_)	Refs.
DSM 70877	15	95.1	18	17.04	22	21.30	8.67	10.69	0.51	0.09	0.11	This work
DSM 70877	15	179.4	19	34.6	21	38	17.5	19.2	0.50	0.10	0.11	[7]
DSM 70877	50	121.9	13.5	16.49	n.a.	n.a.	8.32	n.a.	0.49	0.07	n.a.	[14]
X-33	30	70.7	n.a.	n.a.	n.a.	n.a.	n.a.	n.a.	0.50	n.a.	n.a.	[15]
GS115	30-	102	n.a.	n.a.	n.a.	n.a.	n.a.	n.a.	0.57	n.a.	n.a.	[16]

## Data Availability

Data will be made available upon request.

## References

[1] Araújo D., Ferreira I.C., Torres C.A.V., Neves L., Freitas F. (2020). Chitinous polymers: Extraction from fungal sources, characterization and processing towards value-added applications. J. Chem. Technol. Biotechnol..

[2] Bottin J.H., Swann J.R., Cropp E., Chambers E.S., Ford H.E., Ghatei M.A., Frost G.S. (2016). Mycoprotein reduces energy intake and postprandial insulin release without altering glucagon-like peptide-1 and peptide tyrosine-tyrosine concentrations in healthy overweight and obese adults: A randomised-controlled trial. Br. J. Nutr..

[3] Neyrinck A.M., Catry E., Taminiau B., Cani P.D., Bindels L.B., Daube G., Dessy C., Delzenne N.D. (2019). Chitin–glucan and pomegranate polyphenols improve endothelial dysfunction. Sci. Rep..

[4] Abdel-Mohsen A.M., Jancar J., Massoud D., Fohlerova Z., Elhadidy H., Spotz Z., Hebeish A. (2016). Novel chitin/chitosan-glucan wound dressing: Isolation, characterization, antibacterial activity and wound healing properties. Int. J. Pharm..

[5] Liu Y., Wu Q., Wu X., Algharib S.A., Gong F., Hu J., Luo W., Zhou M., Pan Y., Yan Y. (2021). Structure, preparation, modification, and bioactivities of β-glucan and mannan from yeast cell wall: A review. Int. J. Biol. Macromol..

[6] Tanihiro R., Sakano K., Oba S., Nakamura C., Ohki K., Hirota T., Sugiyama H., Ebihara S., Nakamura Y. (2020). Effects of yeast mannan which promotes beneficial *Bacteroides* on the intestinal environment and skin condition: A randomized, double-blind, placebo-controlled study. Nutrients.

[7] Farinha I., Araújo D., Freitas F. (2019). Optimization of medium composition for production of chitin-glucan complex and mannose-containing polysaccharides by the yeast *Komagataella pastoris*. J. Biotechnol..

[8] Arroyo J., Farkaš V., Sanz A.B., Cabib E. (2016). Strengthening the fungal cell wall through chitin–glucan cross-links: Effects on morphogenesis and cell integrity. Cell. Microbiol..

[9] Sanz A.B., García R., Rodríguez-Peña J.M., Arroyo J. (2018). The CWI pathway: Regulation of the transcriptional adaptive response to cell wall stress in yeast. J. Fungi.

[10] Liu W.-C., Inwood S., Gong T., Sharma A., Yu L.-Y., Zhu P. (2019). Fed-batch high-cell-density fermentation strategies for *Pichia pastoris* growth and production. Crit. Rev. Biotechnol..

[11] Çalık P., Ata Ö., Günes H., Massahi A., Boy E., Keskin A., Öztürk S., Zerze G.H., Özdamar T.H. (2015). Recombinant protein production in *Pichia pastoris* under glyceraldehyde-3-phosphate dehydrogenase promoter: From carbon source metabolism to bioreactor operation parameters. Biochem. Eng. J..

[12] Chagas B., Farinha I., Galinha C.F., Freitas F., Reis M.A.M. (2014). Chitin-glucan complex production by *Pichia (Pichia) pastoris*: Impact of cultivation pH and temperature on polymer content and composition. New Biotechnol..

[13] Gmeiner C., Saadati A., Maresch D., Krasteva S., Frank M., Altmann F., Herwig C., Spadiut O. (2015). Development of a fed-batch process for a recombinant *Pichia pastoris* Δoch1 strain expressing a plant peroxidase. Microb. Cell Fact..

[14] Farinha I., Freitas F., Reis M.A.M. (2017). Implementation of a repeated fed-batch process for the production of chitin-glucan complex by *Pichia pastoris*. New Biotechnol..

[15] Robert J.M., Garcia-Ortega X., Montesinos-Seguí J.L., Freire D.M.G., Valero F. (2019). Continuous operation, a realistic alternative to fed-batch fermentation for the production of recombinant lipase B from *Candida antarctica* under the constitutive promoter PGK in *Pichia pastoris*. Biochem. Eng. J..

[16] Noseda D.G., Recúpero M., Blasco M., Bozzo J., Galvagno M.A. (2016). Production in stirred-tank bioreactor of recombinant bovine chymosin B by a high-level expression transformant clone of *Pichia pastoris*. Protein Expr. Purif..

[17] Araújo D., Freitas F., Sevrin C., Grandfils C., Reis M.A.M. (2017). Co-production of chitin-glucan complex and xylitol by *Komagataella pastoris* using glucose and xylose mixtures as carbon source. Carbohydr. Polym..

[18] D’Anjou M.C., Daugulis A.J. (2000). Mixed-feed exponential feeding for fed-batch culture of recombinant methylotrophic yeast. Biotechnol. Lett..

[19] Günes H., Çalık P. (2016). Oxygen transfer as a tool for fine-tuning recombinant protein production by *Pichia pastoris* under glyceraldehyde-3-phosphate dehydrogenase promoter. Bioprocess Biosyst. Eng..

[20] Luo Z., Miao J., Luo W., Li G., Du Y., Yu X. (2018). Crude glycerol from biodiesel as a carbon source for production of a recombinant highly thermostable b-mannanase by *Pichia pastoris*. Biotechnol. Lett..

[21] Gow N.A.R., Latge J.-P., Munro C.A. (2017). The fungal cell wall: Structure, biosynthesis, and function. Microbiol. Spectr..

[22] Nawawi W.M.F.W., Lee K.-Y., Kontturi E., Bismarck A., Mautner A. (2020). Surface properties of chitin-glucan nanopapers from *Agaricus bisporus*. Int. J. Biol. Macromol..

[23] Hong Y., Ying T. (2019). Characterization of a chitin-glucan complex from the fruiting body of *Termitomyces albuminosus* (Berk.) Heim. Int. J. Biol. Macromol..

[24] Zeynali M., Hatamian-Zarmi A., Larypoor M. (2019). Evaluation of chitin-glucan complex production in submerged culture of medicinal mushroom of *Schizophilum commune*: Optimization and growth kinetic. Iran J. Med. Microbiol..

[25] Galinari E., Sabry D.A., Sassaki G.L., Macedo G.R., Passos F.M.L., Mantovani H.C., Rocha H.A.O. (2017). Chemical structure, antiproliferative and antioxidant activities of a cell wall α-d-mannan from yeast *Kluyveromyces marxianus*. Carbohydr. Polym..

[26] Faustino M., Durão J., Pereira C.F., Pintado M.E., Carvalho A.P. (2021). Mannans and mannan oligosaccharides (MOS) from *Saccharomyces cerevisiae*—A sustainable source of functional ingredientes. Carbohydr. Polym..

[27] Liu Y., Huang G., Lv M. (2018). Extraction, characterization and antioxidant activities of mannan from yeast cell wall. Int. J. Biol. Macromol..

[28] Serba E., Pimenov N., Mochalina P., Overchenko M., Borscheva Y., Sharikov A., Rimareva L. (2020). Production of *Aspergillus oryzae* RCAM 01133 biomass with increased protein and polysaccharides content using by-products of food industry. Agron. Res..

